# Identification of anaerobic bacterial strains by pyrolysis-gas chromatography-ion mobility spectrometry

**DOI:** 10.3389/fbioe.2025.1582565

**Published:** 2025-05-30

**Authors:** Tim Kobelt, Jonas Klose, Rumjhum Mukherjee, Martin Lippmann, Szymon P. Szafranski, Meike Stiesch, Stefan Zimmermann

**Affiliations:** ^1^ Department of Sensors and Measurement Technology, Institute of Electrical Engineering and Measurement Technology, Leibniz University Hannover, Hannover, Germany; ^2^ Department of Prosthetic Dentistry and Biomedical Materials Science, Hannover Medical School, Hannover, Germany; ^3^ Lower Saxony Centre for Biomedical Engineering, Implant Research and Development (NIFE), Hannover, Germany

**Keywords:** biofilms, anaerobic bacteria, identification, pyrolysis, gas chromatography, ion mobility spectrometry, support vector machine

## Abstract

The rapid identification of bacterial pathogens is critical for the early diagnosis of severe clinical conditions, such as sepsis or implant-associated infections, and for the initiation of timely, targeted therapies. This need is particularly acute within the complex oral microbiome, where diverse opportunistic pathogens contribute to a range of local and systemic diseases. While techniques such as phenotypic systems and MALDI-TOF-MS offer faster results, they remain limited by costs, and operational constraints. To address these challenges and cater to the need for rapid identification of bacteria, we present a system for identification and classification of anaerobic bacteria as a first example. This system combines a pyrolyzer, a gas chromatograph and a highly sensitive ion mobility spectrometer. The ion mobility spectrometer has been optimized for coupling with the gas chromatograph and offers simultaneously recording of ion mobility spectra in both ion polarities during one gas chromatographic separation by using two drift tubes arranged in axial configuration. Feasibility has been demonstrated by building a database of fingerprints of eleven isolated reference samples of anaerobic bacteria with clinical relevance. Preliminary experiments have demonstrated that pattern recognition algorithms can predict the genus of isolated bacteria with a precision of up to 97%.

## 1 Introduction

The rapid identification of bacterial pathogens in clinical environments is imperative for the early diagnosis of critical conditions such as sepsis (Brook, 2007). Prompt and accurate detection empowers clinical teams to make evidence-based decisions (Simons and Capraro, 2024) and initiate patient-specific therapeutic strategies, thereby significantly improving clinical outcomes. This need is particularly pronounced in complex microbial ecosystems such as the oral microbiome, where a highly diverse and dynamic community of commensal and pathogenic bacteria coexist. The human oral microbiome plays a critical role in several prevalent and significant health conditions, both local and systemic (Lamont et al., 2018). It comprises of hundreds of physiologically diverse, biofilm-associated microorganisms inhabiting various oral microenvironments, with an even greater diversity of unique genotypes (Baker et al., 2024). Among these, certain species, primarily anaerobes, e.g., *Fusobacterium*, *Porphyromonas*, *Prevotella* and *Veillonella* have been associated with oral infections, including severe, hard-to-treat conditions like peri-implantitis as well as odontogenic infections, which can occasionally become life-threatening (Ghensi et al., 2020).

Given these risks, there is a pressing clinical need for methodologies that enable rapid on-site identification of potential pathogens, allowing them to be distinguished from benign species. This is especially crucial during the early stages of disease, even before symptoms manifest, when the condition is more manageable and responsive to treatment (Dieckow et al., 2024). In these circumstances, a timely bacterial identification is fundamental in optimizing the precise administration of bacteria-specific antibiotics and mitigating the emergence and spread of global antimicrobial resistance (Yan et al., 2024). Furthermore, the rapid taxonomic assignment is a valuable feature of culturomics, which focuses on isolating numerous strains from complex microbial communities, including polymicrobial infections (Huang et al., 2023). These isolates are highly useful for mechanistic molecular studies, supporting the development of preventive and therapeutic strategies against intricate polymicrobial infections.

Traditional methods such as Phenotyping (BioMérieux, Beckman Coulter, BD Diagnostics, Thermo Fisher Scientific), 16S rRNA Gene Sequencing and shotgun whole genome sequencing often require several hours (4–96 h) to yield results, which can delay treatment decisions and negatively impact patient outcomes (Heaton and Bhatti, 2020). While additional methods such as Fourier-transform infrared (FT-IR) spectroscopy (Naumann et al., 1991) and matrix-assisted laser ionization time-of-flight mass spectrometry (MALDI-TOF-MS) (Lay, 2001), are widely used in clinical settings for the rapid identification of bacteria, these systems are dependent on a large database containing curated reference spectra of a large variety of species, less accurate for closely related species and sensitive to sample conditions.

In response to these challenges, the approach presented here aims to provide an alternative rapid method for differentiating bacterial strains with demonstrated feasibility using clinically relevant anaerobic bacterial strains from the human oral microbiome. This approach relies on the use of pyrolysis (Py) in combination with a gas chromatography (GC). In the Py-GC process, bacterial strains, composed of a large number of small molecules such as amino acids, carbohydrates, fatty acids, nucleotides, quinones, vitamins (Watson et al., 1987), are fragmented by thermal energy inside the pyrolyzer, resulting in volatile fragments. These fragments are subsequently separated by GC. Mass spectrometry (MS) is often employed as a detector in these hyphenated systems, providing a second dimension of separation based on the mass-to-charge ratio (m/z). The differentiation of bacterial strains using Py-GC-MS has been demonstrated in previous studies (Meuzelaar and Kistemaker, 1973; Basile et al., 1998; Dworzanski et al., 2005; Melucci et al., 2013; Picó and Barceló, 2020). As an alternative, ion mobility spectrometry (IMS) can be used instead of MS giving a pyrolyzer-gas chromatograph-ion mobility spectrometer (Py-GC-IMS) (Snyder et al., 2004; Schmidt et al., 2004; Prasad et al., 2006; Prasad et al., 2007). IMS offers very high sensitivity at compact size and low cost and also adds a second dimension of separation (ion mobility). In addition, IMS typically operate at ambient pressure and therefore do not require a bulky vacuum system. This allows for mobile and compact instruments to be used on site.

Drift tube IMS with field-switching ion shutters are particularly suited for coupling to a gas chromatograph, as they have a comparatively small effective detector volume in comparison to IMS with beam chopping shutters (Nitschke et al., 2024; Kobelt et al., 2024a). Moreover, they reach significantly higher sensitivity than beam-shopping ion shutters (Bohnhorst et al., 2021). In IMS with field switching ion shutter, the ionized analyte molecules accumulate in the ionization region before being injected into the drift region where they are separated in an homogeneous electric field according to their ion mobility (Jenkins and McGann, 2002). This process is typically repeated every 5–30 ms (Borsdorf and Eiceman, 2006; Borsdorf et al., 2011), which is sufficient for resolving GC peaks even of fast GC with typical peak width of 1–3 s (Korytár et al., 2002). The mobility of an ion can be calculated based on Equation 1 and depends on the ion charge Q , the drift gas density n , the reduced mass of the ion µ , the Boltzmann constant *k*_*b*_, the absolute drift gas temperature T and the collision cross section of the ion and the drift gas molecules σ (Revercomb and Mason, 1975).

$$K=\frac{3}{16}\sqrt{\frac{2\pi }{µk_{B}T}\;\cdot }\frac{Q}{n\sigma }$$ (1)

In order to demonstrate the feasibility of a Py-GC-IMS for the identification of bacteria beyond the previously shown differentiation of, *e.g.*, Gram-positive and Gram-negative bacteria (Snyder et al., 2004), an IMS with significantly improved analytical performance is used here. Since a Py-GC-IMS does not allow the identification of unknown pyrolysis fragments, the differentiation of individual bacteria in this work is based on the unique fragment pattern of the individual bacteria. For this reason, pure reference samples of the bacteria are measured in order to obtain characteristic fingerprints of the respective bacteria and afterwards a classification algorithm is used to differentiate between the four genera represented by the eleven diverse bacterial strains in our database.

## 2 Materials and methods

### 2.1 Measurement system

**FIGURE 1:**
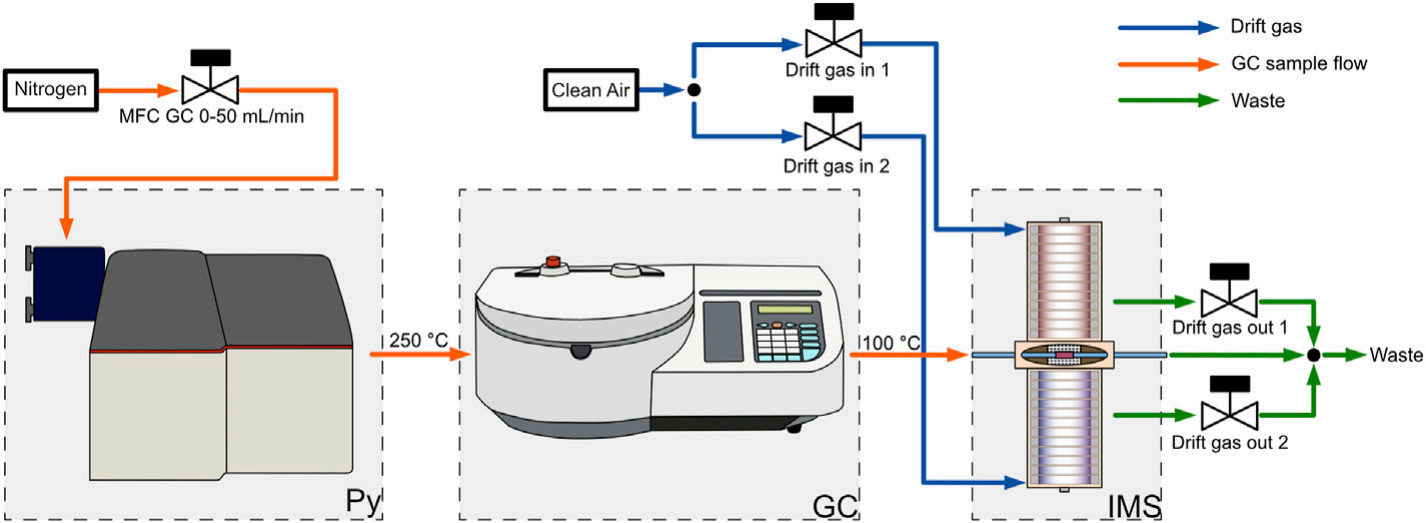
Gas flow chart of the pyrolyzer-gas chromatograph-ion mobility spectrometer (Py-GC-IMS). The GC carrier gas flow is supplied by a mass flow controller and routed through the pyrolyzer into the gas chromatograph. The GC is coupled to the self-build dual polarity ion mobility spectrometer with a special ionization region optimized for coupling with gas chromatography. Clean, dry air is used as the drift gas. The drift gas inlets and outlets are also controlled by mass flow controllers.

**FIGURE 2:**
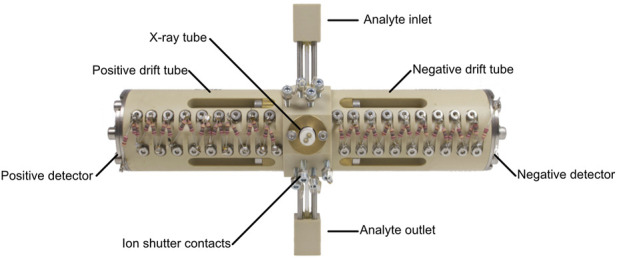
Photo of the self-build dual polarity ion mobility spectrometer with two drift tubes in axial configuration.

The measurement system, shown in Figure 1, contains a commercial pyrolysis unit 6,200 Pyroprobe (CDS Analytics, United States) with the “DISC”-Sample Chamber. This chamber is used to inject quartz sample holders with a maximum sample volume of approximately 5 µL. The pyrolysis chamber is resistively heated, enabling different temperatures and temperature programs. The pyrolysis products are injected into the liner of the Gas Chromatograph 200 (Ellutia, United Kingdom) which is connected via a heated transfer capillary (250°C). The gas flow through the pyrolyzer, also giving the GC carrier gas flow is controlled by a mass flow controller EL-Flow-Prestige (Bronkhorst, Netherlands) instead of the integrated pressure regulator of the GC. A 30 m RXI-5 ms GC column with ID: 530 µm and d_f_: 1.5 µm (Restek, United States) is used to separate the fragments. The transfer line from the GC to the IMS is heated to 100°C. The IMS, shown in Figure 2, consists of two axially arranged drift tubes (Lippmann et al., 2020a) for simultaneously detecting both ion polarities during one GC cycle. The drift tubes are constructed from polyether ether ketone (PEEK), and stainless steel drift rings (Kirk and Zimmermann, 2015) with an effective drift length of 87 mm. Purified dry air is used as drift gas for both drift tubes and is supplied by mass flow controllers IQ+ (Bronkhorst, Netherlands). With an effective detector volume auf just 360 µL (Kobelt et al., 2024a; Kirk et al., 2022), the ionization region is optimized for coupling with a gas chromatograph. The optimized flow geometry ensures that the IMS can be operated at ambient temperature, while only heating the transfer capillary to the IMS is required. An extended field switching shutter is integrated to maximize sensitivity (Kirk et al., 2020). A self-built 600 V power supply and MOSFET half bridges are used to generate the short 200 µs voltage pulses required for the ion shutter. An X-ray source SCXT0829 (Sunje, South Korea) is used to ionize the sample via atmospheric pressure chemical ionization (APCI) (Spangler and Carrico, 1983; Pershenkov et al., 2006). The required electronics for the acceleration voltage and filament supply of the X-ray source are also self-built. For the drift voltage, two HCE 7–12,500 (FuG, Germany) are used in combination with an RC-lowpass filter (2 nF and 2.4 MΩ) to reduce drift voltage ripple. To convert the ion current into a voltage, a self-built transimpedance amplifier with a gain of 5 GΩ and a bandwidth of 25 kHz is used at each detector of the drift tube (Cochems et al., 2014). These two signals are then digitized simultaneously at 250 kSamples per second with 16-bit resolution with our self-built FPGA-based isolated data acquisition (Kobelt et al., 2024b; Lippmann et al., 2020b). The data is digitally low-pass filtered at 25 kHz and then 16 IMS spectra are averaged before saving the spectra to the measurement file to increase the signal-to-noise ratio of the IMS and reduce file size. The data acquisition system also controls the entire measurement setup and records all parameters. To calculate the ion mobilities the pressure inside the drift tube and the ambient temperature are measured during all measurements with a combined pressure, temperature and humidity sensor BME280 (Bosch, Germany). This allows for matching IMS peak positions over long measurement durations. To synchronize the GC, the Py and the IMS, the Py toggles a TTL output at the start of the pyrolysis and the GC and IMS start their measurement cycle. Although mainly self-built electronics was used in this setup, any commercially available equipment that provides the electric fields and the switching times given by Table 1 may be used instead.

**TABLE 1:** Operating parameters IMS.

Effective drift voltage	6.4 kV
Effective drift length	87 mm
Drift region diameter	21 mm
Injection field	240 V/mm
Injection time	600 µs
Compensation field	−26 V/mm
IMS temperature	25°C
Dew point carrier and drift gas	−98°C
Drift gas flow rate in	150 mL/min
Drift gas flow rate out	100 mL/min
IMS period	30 ms
IMS averages	16
IMS pressure	1,010 hPa
X-Ray accelerating voltage	4950 V
X-Ray emission current	5.4 µA

### 2.2 Strains and culture conditions

The study is focused on eleven anaerobic bacterial strains, summarized in Table 2, representing three classes, four genera, and seven species. All these species are associated with oral infections, including peri-implant diseases. The collection includes both reference type strains and recent clinical isolates.

**TABLE 2:** Bacterial strains and their origin used in the study.

SPS number	Class	Genus	Species	Type strain	Other names	Clinical origin	Source
453	Bacteroidia	*Porphyromonas*	*gingivalis*	Yes	DSM 20709	Human gingival sulcus	DSMZ, Braunschweig, Germany
803	Bacteroidia	*Porphyromonas*	*gingivalis*		DSM 28984, HG 66	Human oral cavity	DSMZ, Braunschweig, Germany
21	Bacteroidia	*Prevotella*	*buccae*			Human peri-implantitis	Own isolate
577	Bacteroidia	*Prevotella*	*denticola*			Human peri-implantitis	Own isolate
457	Bacteroidia	*Prevotella*	*intermedia*	Yes	DSM 20706	Empyema	DSMZ, Braunschweig, Germany
447	Fusobacteriia	*Fusobacterium*	*nucleatum* subsp. *nucleatum*	Yes	DSM 15643	Cervico-facial lesion	DSMZ, Braunschweig, Germany
527	Fusobacteriia	*Fusobacterium*	*nucleatum* subsp. *polymorphum*	Yes	DSM 20482	Inflamed gingiva	DSMZ, Braunschweig, Germany
805	Fusobacteriia	*Fusobacterium*	*nucleatum* subsp. *vincentii*	Yes	CCUG 37843	Human periodontal pocket	CCUG, Gothenburg, Sweden
13	Negativicutes	*Veillonella*	*dispar*			Human peri-implantitis	Own isolate
884	Negativicutes	*Veillonella*	*parvula*		PK1910	Subgingival dental plaque	Nicholas Jakubovics, Newcastle University, Newcastle, United Kingdom
513	Negativicutes	*Veillonella*	*parvula*			Human peri-implantitis	Own isolate

The bacterial strains were cultured on Fastidious Anaerobe Agar (FAA; CE, LAB090/NCM2020A, LabM/Neogen, United Kingdom) supplemented with 5% defibrinated sheep blood (SR0051E, ThermoScientific, United States). Cultivation was conducted under anaerobic conditions (80% N_2_, 10% CO_2_, and 10% H_2_) using an anaerobic chamber (Concept 400 Anaerobic Workstation; Ruskinn Technology Ltd., Leeds, United Kingdom) at 37°C for 3 days.

Bacterial biomass was harvested with sterile inoculation loops (10 μL, #86.1562.050, Sarstedt, Germany) and suspended in oxygen-free Dulbecco’s Phosphate-Buffered Saline (D8537-500 mL, Sigma-Aldrich, Germany). The suspension was centrifuged at 4,000 rpm for 10 min using an 5430G centrifuge (#EP5427000610, Eppendorf, Germany) to obtain cell pellets. The supernatant was carefully discarded, and the cell pellets were stored at −20°C for future use.

### 2.3 Measurements

For each measurement, a new quartz glass sample tube is cleaned at 1,100°C for 10 s. In order to measure a reproducible amount of biomass in each experiment, the biomass of the corresponding sample is dissolved in deionized water in a mass ratio of 1:100 and mixed for 10 s in a vortex mixer. 2.5 µL of the mixtures are pipetted into the sample tube, which results in 25 ng biomass in each measurement. A buffer is not used at this point because the bacteria are immediately dried and then pyrolyzed and any possible contamination of the sample should be avoided. A temperature of 150°C for 120 s proved to be a sufficient drying step to remove the excess water content of the sample. The compounds released in the cleaning and drying step are vented out of the pyrolyzer via the integrated eight-port valve and are not transferred to the GC. The sample tube remains in the pyrolyzer after the drying step and pyrolysis at 700°C for 10 s is started. These pyrolysis settings were derived from literature, where temperatures for similar pyrolysis setups and bacterial samples range from 650°C to 800°C (Prasad et al., 2006; Schmidt et al., 2004; Sam and Wampler, 2021). Different temperatures and pyrolysis profiles have not been explored yet. The fastest heating rate of the pyrolyzer of 20°C/ms (according to the datasheet) is selected. The interface of the pyrolysis chamber and the injector temperature are maintained at 200°C and the eight-port valve at 250°C. The temperature program of the GC starts simultaneously with the start of the pyrolysis run. The GC holds a temperature at 60°C for 5 min and then ramps at 15°C/min to 250°C, which is held for another 5 min. Nitrogen is used as carrier gas at a flow rate of 10 mL/min. In total, 48 measurements were performed for the bacterial strains shown in Table 2. Prior to each biomass measurement, a blank measurement is performed with the same cleaned quartz sample holder using the measurement protocol of the bacterial strains.

**FIGURE 3:**
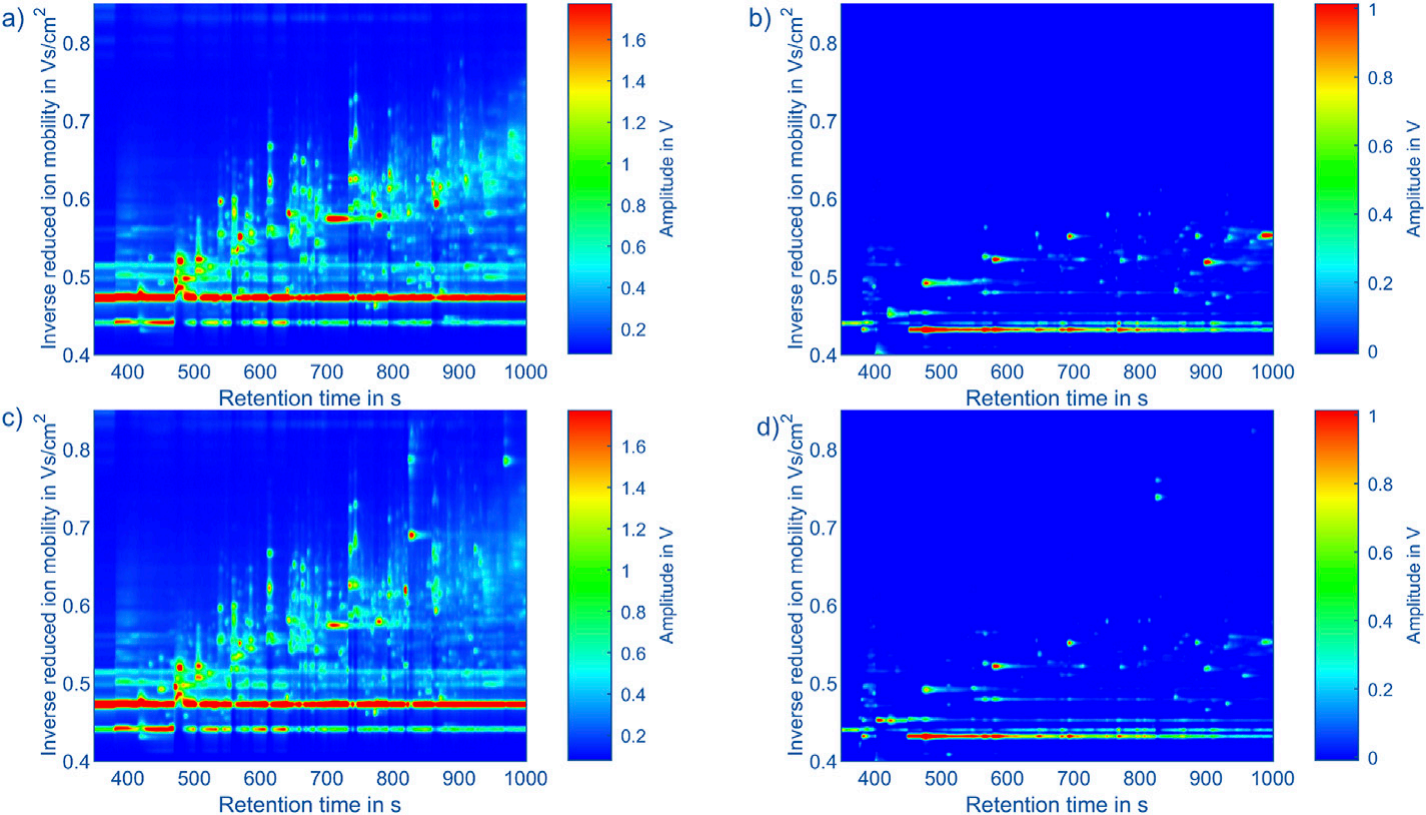
Topographic plots of the measurement data of: **(a)** *Porphyromonas gingivalis* (453) in positive polarity, **(b)** *Porphyromonas gingivalis* (453) in negative polarity, **(c)** *Fusobacterium nucleatum* subsp. *vincentii* (805) in positive polarity, **(d)** *Fusobacterium nucleatum* subsp. *vincentii* (805) in negative polarity. The x-axis shows the retention time of the gas chromatograph and the y-axis the inverse reduced ion mobility. The color indicates the amplitude of the detector signal of the IMS. The color scaling within a polarity is identical.

For example, topographic plots of the measurement data in positive and negative polarity of *Fusobacterium nucleatum* subsp. *vincentii* (805) and *Porphyromonas gingivalis* (453) are shown in Figure 3. In this topographic representation the GC retention time is given in seconds on the x-axis and the inverse reduced ion mobility in Vs/cm^2^ on the y-axis. The color value encodes the ion current reaching the detector of the IMS. To obtain these mobility values, the drift time *t*_d_ of the IMS is converted according to Equation 2 using the recorded pressure *p*, temperature *T*, effective drift voltage *U*_d_ and effective drift length *L*_d_ to align the peaks in the IMS dimension. This mitigates ambient pressure and temperature variations during one GC cycle and over the timespan of several days. Considering the deviating electric field strength in the ion shutter region and aperture grid region, an effective drift voltage and drift length are calculated based on the drift field strength (according Supplementary Formula 1-7).

$$\frac{1}{K_{0}}=\frac{U_{d}\cdot t_{d}}{L_{d}^{2}}\cdot \frac{T}{T_{0}}\;\cdot \frac{p_{0}}{p}$$ (2)

**FIGURE 4:**
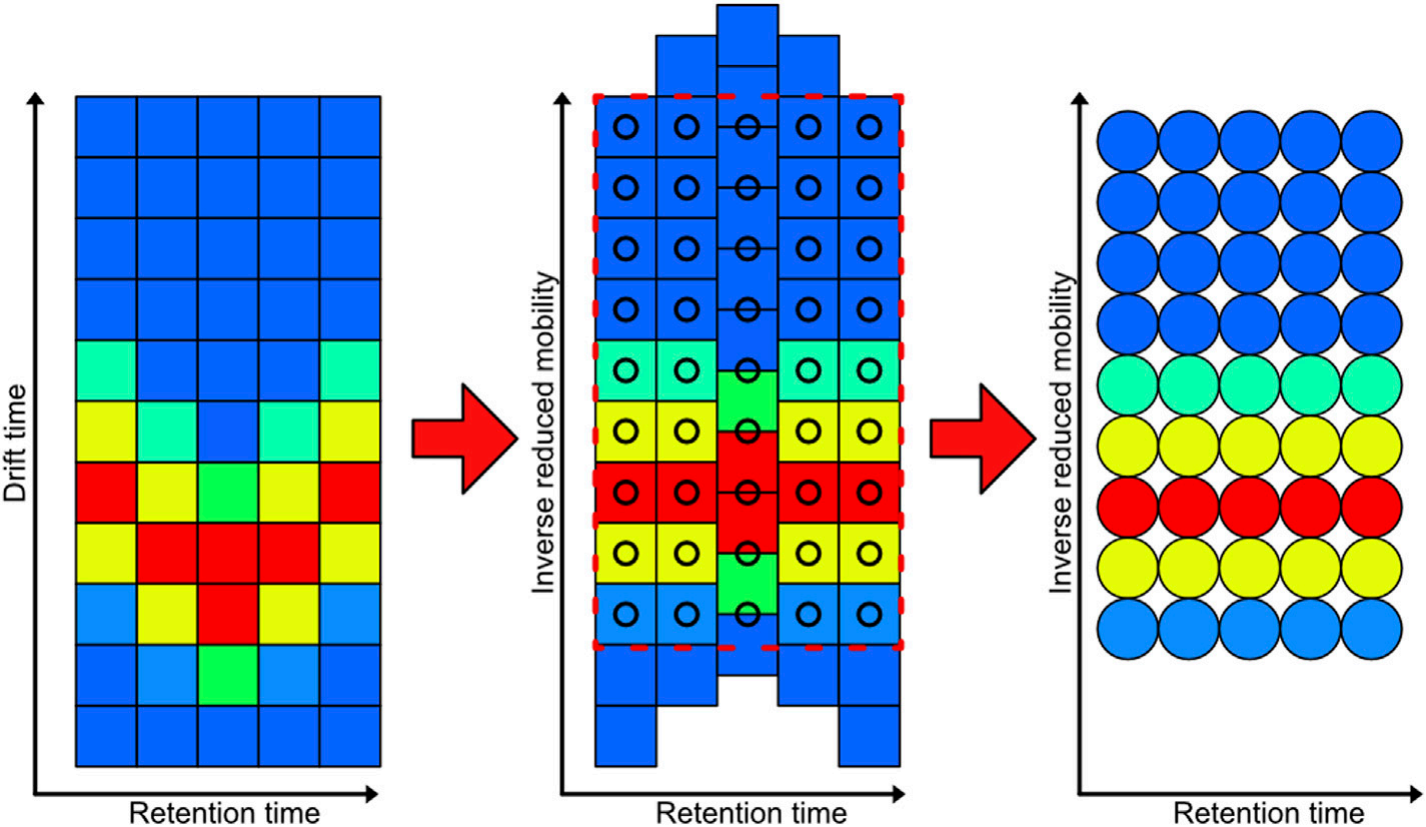
Demonstration of the interpolation process of the GC-IMS data from each measurement to a new fixed pixel grid. The color indicates the intensity of each measurement point, which corresponds to a pixel value in the resulting image.

Each measurement of the whole data set is interpolated on a fixed grid of 3,000 × 3,000 data points by using a workflow implemented in Mathworks MATLAB shown in Figure 4. The first step in the process is to convert the data from drift time to inverse reduced mobilities. This step shifts the IMS spectra towards each other. Next, the data is trimmed and interpolated to a new fixed pixel raster which is consistent over alle measurements in the dataset. This step results in pixel images that have the same retention time and inverse recued mobility for each pixel over the entire data set.

## 3 Results and discussion

**FIGURE 5:**
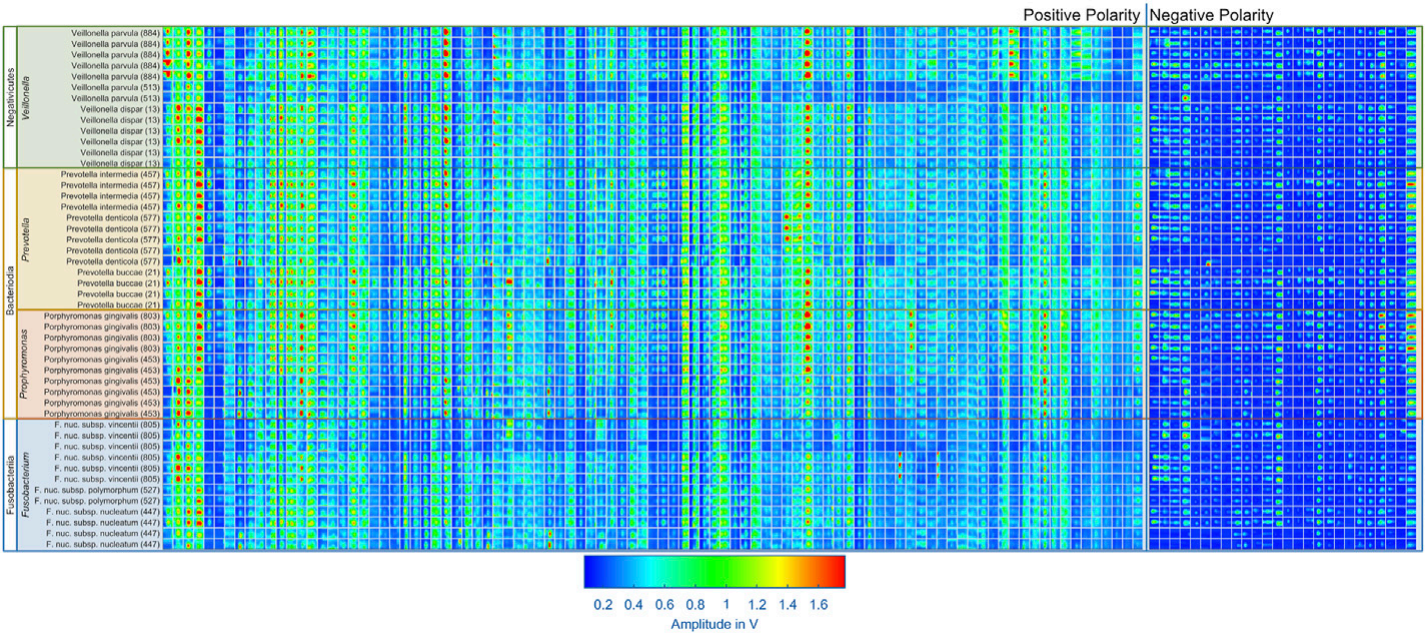
Overview of all 95 selected peaks recorded in positive IMS polarity and 26 selected peaks recoded in negative IMS polarity for each measurement in the dataset. Each box shows the extracted peak from the corresponding measurement displayed on the y-axis. The color indicates the amplitude of the IMS detector signal, ranging from blue to green to red. The peaks are sorted on the x-axis from left to right according to their retention time.

When comparing the measurements from different bacterial strains, as exemplarily shown in Figure 3, a lot of similarities are visible in the positive and negative spectra. However, certain peaks seem exclusive to certain bacterial strains, particularly in the positive polarity. Additionally, the intensities of peaks vary depending on the bacterial strain. While these differences may originate from the bacterial strains, they may also result from contaminants present in certain samples. Another contributing factor could be variations in handling of samples. Despite efforts to control these factors by preparing a blank measurement before each sample analysis, they cannot be completely ruled out. To get an overview of the peaks in all measurements, 95 peaks in the positive polarity and 26 peaks in the negative polarity were manually selected. During this process, fluctuation in the retention times of the early eluting peaks were observed, likely due to unstable starting temperatures of the GC. However, retention times of the later eluting peaks remained stable. To address this issue, the peak positions in retention time were adjusted using linear interpolation of the peak offsets between two peaks consistently present in each measurement. A square mask of 20 × 20 data points is placed at each corrected peak position to extract the peaks in each measurement. The results are shown in Figure 5 for the positive and negative polarity. Given the comparatively low signal intensities in the negative polarity and the large number of peaks in the positive polarity relative to the number of measurements, only the 95 peaks from the positive polarity are considered for the classification algorithm. In future measurement campaigns with a significantly larger number of measurements and bacterial strains, the same methods can be used including the data from the negative polarity.

**FIGURE 6:**
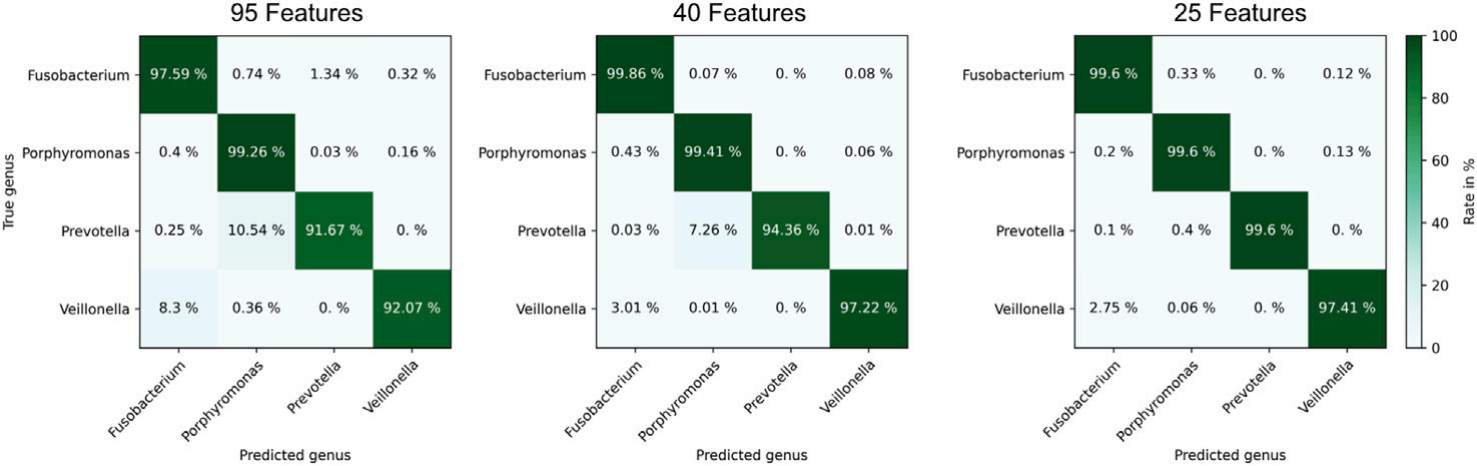
Confusion matrix of the predicted genus by the classification model for all 95 peaks from the positive polarity (left), the 40 most relevant peaks from the positive polarity (middle) and only the 25 most relevant peaks from the positive polarity (right). Each matrix entry represents the percentage with which the sample (y-axis) was assigned to a specific label (x-axis). The diagonal corresponds to the correct assignments.

**FIGURE 7:**
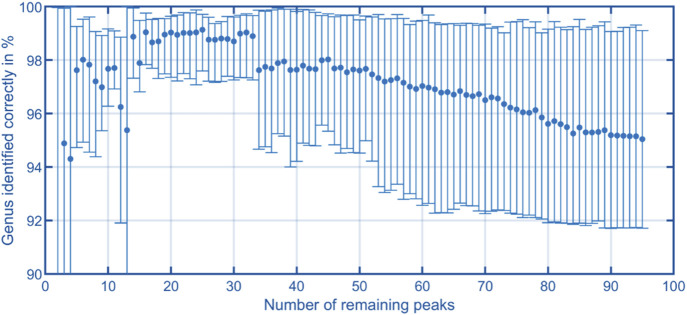
Trend of the correct detection rate, derived from the average of the diagonals of the confusion matrices, plotted against the number of most relevant peaks. Error bars represent the minimum and maximum values of the diagonal for each confusion matrix.

**FIGURE 8:**
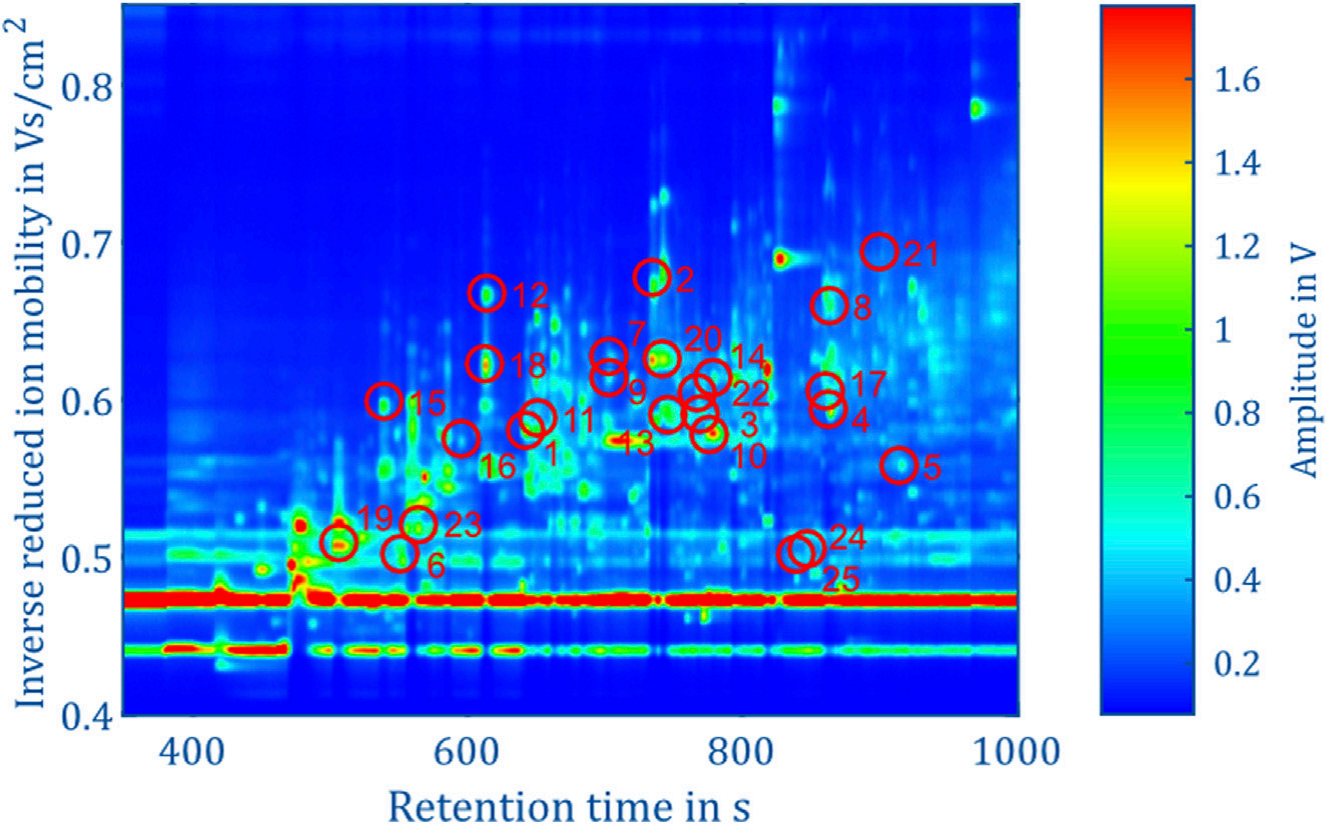
Topographic plot of the experimental data in positive IMS polarity of *Fusobacterium nucleatum* subsp. *vincentii* (805) from Figure 3, highlighting the 25 most relevant peaks along with their order of relevance in the corresponding trained model.

The aim of this work is to classify the bacterial strains representing major opportunistic peri-implant pathogens at the genus level. For demonstrating feasibility, this work is focused on a representative collection of eleven bacterial strains which are assigned to one of the three classes and four genera: Fusobacteriia/*Fusobacterium*: 12, Bacteroidia/*Porphyromonas*: 10, Bacteroidia/*Prevotella*: 13, Negativicutes/*Veillonella*: 13. To classify the genera of present in an unknown sample, Support Vector Machines (Noble, 2006) was used. The dataset containing the peaks of positive polarity of the 48 measurements shown in Figure 5 was used as the model’s input. The input features used for training were obtained by integrating the signal intensities inside each square mask, representing each peak with a single scalar value. Before training the model, the values are z-score normalized. The genus is used as the training labels. The training process involved the use of scikit-learn 1.6 in Python (Pedregosa et al., 2011), with the “linearSVC” model configured with its default settings. This implementation uses a quadratic hinge loss function and a linear kernel function. Using the “SVC” function from scikit-learn, various kernel functions were evaluated with their default settings on the complete dataset of 95 positive peaks. The results indicate that in this case a linear kernel function provided the best classification performance. The confusion matrices for the alternative kernel functions are included in the Supplementary Information 1–4. It is worth noting that non-linear kernel functions may offer better results by systematically optimizing their model parameters. Such optimization will be part of future work. A k-fold cross-validation approach was used to validate the trained models, whereby the data was randomized and divided into five sets. Four sets were allocated for model training, while the fifth set served as the validation set. After five steps, the data set is randomized again, and the process is repeated. For each validation, the number of times the genus of the unknown samples is predicted correctly is recorded. After training 1,000 models, a confusion matrix is generated in which the frequencies of the assignment of the genera by the model, see Figure 6 (left). Using all 95 selected peaks of the positive IMS polarity, *Fusobacterium* and *Porphyromonas* are correctly classified in over 97% of the cases. Only *Prevotella* is misclassified as Porphyromonas about 10% of the time and *Veillonella* is misclassified as *Fusobacterium* 8.5% of the time. The relevance of each peak in identifying unknown samples can be assessed by analyzing the coefficients of the fitted model. After each of the 1,000 runs, the peaks are sorted according to their relevance and the least relevant peak is removed from the dataset. Then, the training and validation are repeated until only two peaks remain in the dataset for identification. Figure 7 shows the mean percentage of the diagonal of the confusion matrix for each number of remaining peaks in the dataset. In addition, the minimal and maximal percentage of correct identification are shown by the error bars in Figure 7. The model’s performance consistently improves as the dataset is reduced from the original 95 peaks to the 34 most relevant peaks. It reaches its optimal performance when using between 32 and 20 peaks, but beyond this range, the dataset appears to lack sufficient information for precise identification of samples. Confusion matrices for the 40 most relevant peaks (middle) and 25 most relevant peaks (right) are shown in Figure 6. It can be observed that the prediction of *Fusobacterium* and *Porphyromonas* remains high and is almost independent of the number of peaks used for classification, as long as the number of peaks used falls between 95 and 20. Meanwhile, classification accuracy for *Veillonella* improves steadily, with misidentification rates decreasing to roughly 2.5% when using 25 features. *Prevotella* shows best improvement with limiting the number of most relevant peaks, which improves identification rates from 91% to 99.5%. Figure 8 shows the 25 most relevant peaks for the best performing model, along with their order of relevance within this trained model. In addition, an overview of these 25 peaks across all measured samples is included in the Supplementary Information 5. Notably, the most relevant peaks are distributed across the full retention time range, from 500 to 900 s. Furthermore, the most relevant peaks have comparatively low intensities in the spectra. Furthermore, due to the chemical ionization of analytes in the IMS, the peaks may be protonated monomers or proton-bound dimers of the same analyte or product ions which only occur when two substances coelute from the GC. This means that there could ultimately be fewer analytes relevant for classification than the number of peaks indicates. Using the 25 most relevant peaks from the dataset of the different bacterial strains, an accuracy of over 97% can be achieved for taxonomic classification of bacteria up to the genus level. It is important to note that this involves only the identification of isolated bacterial strains. In real applications, combinations of various bacteria and complex background matrices may lead to interactions during pyrolysis and masking effects in IMS. Therefore, at this stage, we can only demonstrate the basic feasibility of the technology for rapid identification of selected clinical isolates. Subsequent scientific investigations need to explore the system’s robustness against background media, limits of detection, and matrix effects in complex samples.

## 4 Conclusion

In this work, we use a dual polarity IMS with an optimized ionization region for coupling with gas chromatography with front-end pyrolyzer. The IMS enables simultaneous detection of relevant signatures in both ion polarities in a single GC run. Despite the small dataset, this work shows feasibility of differentiating clinical isolates of anaerobic bacteria by the genus level using a pyrolysis-gas chromatography-ion mobility spectrometry. By selecting the 25 most relevant peaks of the GC-IMS data, the four genera, *Fusobacterium*, *Porphyromonas*, *Veillonella*, and *Prevotella*, can be identified with 97% accuracy using the experimental data.

It is worth noting that Py-GC-IMS has a much broader perspective in clinical microbiology applications beyond the contextualization within the oral microbiome presented here. The oral microbiome, in particular the anaerobic bacterial strains investigated here are considered as a first example to show general feasibility. The representative bacteria used in our study are commonly found in oral biofilms and were selected to demonstrate the basic capability to differentiate between individual genera. Currently, our focus for future analysis of polymicrobial infections is on the analysis of clinical isolates. However, very complex biofilms, such as oral microbial communities, pose major additional challenges and require further methodological development beyond the scope of this work. Therefore, feasibility of differentiating selected clinical isolates by Py-GC-IMS cannot yet be transferred to real clinical applications with complex biofilms. Future analyses involving different bacteria from various sources and in complex backgrounds will further challenge this approach using Py-GC-IMS for rapid on-site identification of bacteria. Moreover, on-site detection would require the miniaturization of the entire system, which is an engineering task conceivable with the selected technologies.

## Data Availability

The raw data supporting the conclusions of this article will be made available by the authors, without undue reservation.

## References

[B1] Baker J. L. Mark Welch J. L. Kauffman K. M. McLean J. S. He X. (2024). The oral microbiome: diversity, biogeography and human health. Nat. Rev. Microbiol. 22 (2), 89–104. 10.1038/s41579-023-00963-6 37700024 PMC11084736

[B2] Basile F. Beverly M. B. Voorhees K. J. Hadfield T. L. (1998). Pathogenic bacteria: their detection and differentiation by rapid lipid profiling with pyrolysis mass spectrometry. TrAC Trends Anal. Chem. 17 (2), 95–109. 10.1016/s0165-9936(97)00103-9

[B3] Bohnhorst A. Kirk A. T. Zimmermann S. (2021). Toward compact high-performance ion mobility spectrometers: ion gating in ion mobility spectrometry. Anal. Chem. 93 (15), 6062–6070. 10.1021/acs.analchem.0c04140 33825452

[B4] Borsdorf H. Eiceman G. A. (2006). Ion mobility spectrometry: principles and applications. Appl. Spectrosc. Rev. 41 (4), 323–375. 10.1080/05704920600663469

[B5] Borsdorf H. Mayer T. Zarejousheghani M. Eiceman G. A. (2011). Recent developments in ion mobility spectrometry. Appl. Spectrosc. Rev. 46 (6), 472–521. 10.1080/05704928.2011.582658

[B6] Brook I. (2007). Anaerobic infections. Boca Raton: CRC Press.

[B7] Cochems P. Kirk A. Zimmermann S. (2014). In-circuit-measurement of parasitic elements in high gain high bandwidth low noise transimpedance amplifiers. Rev. Sci. Instrum. 85 (12), 124703. 10.1063/1.4902854 25554310

[B8] Dieckow S. Szafrański S. P. Grischke J. Qu T. Doll-Nikutta K. Steglich M. (2024). Structure and composition of early biofilms formed on dental implants are complex, diverse, subject-specific and dynamic. NPJ Biofilms Microbiomes 10 (1), 155. 10.1038/s41522-024-00624-3 39719447 PMC11668855

[B9] Dworzanski J. P. Tripathi A. Snyder A. P. Maswdeh W. M. Wick C. H. (2005). Novel biomarkers for Gram-type differentiation of bacteria by pyrolysis–gas chromatography–mass spectrometry. J. Anal. Appl. Pyrolysis 73 (1), 29–38. 10.1016/j.jaap.2004.09.003

[B10] Ghensi P. Manghi P. Zolfo M. Armanini F. Pasolli E. Bolzan M. (2020). Strong oral plaque microbiome signatures for dental implant diseases identified by strain-resolution metagenomics. NPJ Biofilms Microbiomes 6 (1), 47. 10.1038/s41522-020-00155-7 33127901 PMC7603341

[B11] Heaton P. R. Bhatti M. M. (2020). “Systems for identification of bacteria and fungi,” in ClinMicroNow (Wiley), 1–22.

[B12] Huang Y. Sheth R. U. Zhao S. Cohen L. A. Dabaghi K. Moody T. (2023). High-throughput microbial culturomics using automation and machine learning. Nat. Biotechnol. 41 (10), 1424–1433. 10.1038/s41587-023-01674-2 36805559 PMC10567565

[B13] Jenkins A. McGann W. (2002). Enhancements to ion mobility spectrometers. US 6,765,198 B2.

[B14] Kirk A. T. Küddelsmann M. J. Bohnhorst A. Lippmann M. Zimmermann S. (2020). Improving ion mobility spectrometer sensitivity through the extended field switching ion shutter. Anal. Chem. 92 (7), 4838–4847. 10.1021/acs.analchem.9b04259 32159336

[B15] Kirk A. T. Küddelsmann M. J. Zimmermann S. (2022). Ultrasensitive ion source for drift tube ion mobility spectrometers combining optimized sample gas flow with both chemical ionization and direct ionization. Anal. Chem. 94, 9960–9969. 10.1021/acs.analchem.2c00955 35793469

[B16] Kirk A. T. Zimmermann S. (2015). Pushing a compact 15 cm long ultra-high resolution drift tube ion mobility spectrometer with R = 250 to R = 425 using peak deconvolution. Int. J. Ion. Mobil. Spectrom. 18 (1-2), 17–22. 10.1007/s12127-015-0166-z

[B17] Kobelt T. Lippmann M. Nitschke A. Kielhorn L. Zimmermann S. (2024b). An open source isolated data acquisition with trigger pulse generation for ion mobility spectrometry. HardwareX 20, e00600. 10.1016/j.ohx.2024.e00600 39553919 PMC11565032

[B18] Kobelt T. Lippmann M. Wuttke J. Wessel H. Zimmermann S. (2024a). Influence of ionization volume and sample gas flow rate on separation power in gas chromatography-ion mobility spectrometry. J. Chromatogr. A 1713, 464506. 10.1016/j.chroma.2023.464506 37983986

[B19] Korytár P. Janssen H.-G. Matisová E. Brinkman U. A. (2002). Practical fast gas chromatography: methods, instrumentation and applications. TrAC Trends Anal. Chem. 21 (9-10), 558–572. 10.1016/s0165-9936(02)00811-7

[B20] Lamont R. J. Koo H. Hajishengallis G. (2018). The oral microbiota: dynamic communities and host interactions. Nat. Rev. Microbiol. 16 (12), 745–759. 10.1038/s41579-018-0089-x 30301974 PMC6278837

[B21] Lay J. O. (2001). MALDI-TOF mass spectrometry of bacteria. Mass Spectrom. Rev. 20 (4), 172–194. 10.1002/mas.10003 11835305

[B22] Lippmann M. Kirk A. T. Hitzemann M. Zimmermann S. (2020a). Compact and sensitive dual drift tube ion mobility spectrometer with a new dual field switching ion shutter for simultaneous detection of both ion polarities. Anal. Chem. 92 (17), 11834–11841. 10.1021/acs.analchem.0c02166 32786212

[B23] Lippmann M. Kirk A. T. Hitzemann M. Zimmermann S. (2020b). IMS Instrumentation I: isolated data acquisition for ion mobility spectrometers with grounded ion sources. Int. J. Ion. Mobil. Spectrom. 23, 69–74. 10.1007/s12127-020-00260-5

[B24] Melucci D. Fedi S. Locatelli M. Locatelli C. Montalbani S. Cappelletti M. (2013). Application of pyrolysis-gas chromatography-mass spectrometry and multivariate analysis to study bacteria and fungi in biofilms used for bioremediation. Curr. Drug Targets 14 (9), 1023–1033. 10.2174/1389450111314090011 23721185

[B25] Meuzelaar H. L. Kistemaker P. G. (1973). Technique for fast and reproducible fingerprinting of bacteria by pyrolysis mass spectrometry. Anal. Chem. 45 (3), 587–590. 10.1021/ac60325a051 4586927

[B26] Naumann D. Helm D. Labischinski H. (1991). Microbiological characterizations by FT-IR spectroscopy. Nature 351 (6321), 81–82. 10.1038/351081a0 1902911

[B27] Nitschke A. Hitzemann M. Winkelholz J. Kirk A. T. Lippmann M. Thoben C. (2024). A hyper-fast gas chromatograph coupled to an ion mobility spectrometer with high repetition rate and flow-optimized ion source to resolve the short chromatographic peaks. J. Chromatogr. A 1736, 465376. 10.1016/j.chroma.2024.465376 39277980

[B28] Noble W. S. (2006). What is a support vector machine? Nat. Biotechnol. 24 (12), 1565–1567. 10.1038/nbt1206-1565 17160063

[B29] Pedregosa F. Varoquaux G. Gramfort A. Michel V. Thirion B. Grisel O. (2011). Scikit-learn: machine learning in Python. J. Mach. Learn. Res. 12 (85), 2825–2830. 10.5555/1953048.2078195

[B30] Pershenkov V. S. Tremasov A. D. Belyakov V. V. Razvalyaev A. U. Mochkin V. S. (2006). X-ray ion mobility spectrometer. Microelectron. Reliab. 46 (2-4), 641–644. 10.1016/j.microrel.2005.07.003

[B31] Picó Y. Barceló D. (2020). Pyrolysis gas chromatography-mass spectrometry in environmental analysis: focus on organic matter and microplastics. TrAC Trends Anal. Chem. 130, 115964. 10.1016/j.trac.2020.115964

[B32] Prasad S. Pierce K. M. Schmidt H. Rao J. V. Güth R. Bader S. (2007). Analysis of bacteria by pyrolysis gas chromatography-differential mobility spectrometry and isolation of chemical components with a dependence on growth temperature. Analyst 132 (10), 1031–1039. 10.1039/b705929a 17893807

[B33] Prasad S. Schmidt H. Lampen P. Wang M. Güth R. Rao J. V. (2006). Analysis of bacterial strains with pyrolysis-gas chromatography/differential mobility spectrometry. Analyst 131 (11), 1216–1225. 10.1039/b608127d 17066190

[B34] Revercomb H. E. Mason E. A. (1975). Theory of plasma chromatography/gaseous electrophoresis. Review. Anal. Chem. 47 (7), 970–983. 10.1021/ac60357a043

[B35] Sam K. D. Wampler T. P. (2021). Analytical pyrolysis handbook. 3rd ed. Boca Raton: CRC Press.

[B36] Schmidt H. Tadjimukhamedov F. Mohrenz I. V. Smith G. B. Eiceman G. A. (2004). Microfabricated differential mobility spectrometry with pyrolysis gas chromatography for chemical characterization of bacteria. Anal. Chem. 76 (17), 5208–5217. 10.1021/ac0497611 15373463

[B37] Simons C. C. Capraro G. A. (2024). Barriers to implementation of rapid identification and antimicrobial susceptibility testing technologies in the clinical microbiology laboratory: an American perspective. J. Antimicrob. Chemother. 79 (Suppl. 1), i32–i36. 10.1093/jac/dkae279 39298360 PMC11412235

[B38] Snyder A. P. Dworzanski J. P. Tripathi A. Maswadeh W. M. Wick C. H. (2004). Correlation of mass spectrometry identified bacterial biomarkers from a fielded pyrolysis-gas chromatography-ion mobility spectrometry biodetector with the microbiological gram stain classification scheme. Anal. Chem. 76 (21), 6492–6499. 10.1021/ac040099i 15516146

[B39] Spangler G. E. Carrico J. P. (1983). Membrane inlet for ion mobility spectrometry (plasma chromatography). Int. J. Mass Spectrom. Ion Phys. 52 (2-3), 267–287. 10.1016/0020-7381(83)85048-7

[B40] Watson J. D. Hopkins N. H. Roberts J. W. Weiner A. M. Steitz J. A. (1987). Molecular biology of the gene. 4. ed. Menlo Park, Calif: Benjamin-Cummings.

[B41] Yan B. Zeng L. Lu Y. Li M. Lu W. Zhou B. (2024). Rapid bacterial identification through volatile organic compound analysis and deep learning. BMC Bioinforma. 25 (1), 347. 10.1186/s12859-024-05967-4 PMC11539783 39506632

