# Supplementary material for: Identification of anaerobic bacterial strains by pyrolysis-gas chromatography-ion mobility spectrometry
Source: Front Bioeng Biotechnol. 2025 May 30;13:1582565. doi: 10.3389/fbioe.2025.1582565 (PMC12163022; doi:10.3389/fbioe.2025.1582565)
Supplement: Supplementary file 1 [file DataSheet1.docx]

Supplementary Material

# Supplementary Data

$E_{\mathrm{Drift}}=\frac{U_{\mathrm{Drift}}}{L_{\mathrm{Drift}}}$ (1)

$L_{\mathrm{In}j_{\mathrm{eff}}}=L_{\mathrm{Inj}}\cdot\frac{E_{\mathrm{Drift}}}{E_{\mathrm{Inj}}}$ (2)

$L_{G1G2_{\mathrm{eff}}}=L_{G1G2}\cdot\frac{E_{\mathrm{Drift}}}{E_{G1G2}}$ (3)

$L_{G2G3_{\mathrm{eff}}}=L_{G2G3}\cdot\frac{E_{Drift}}{E_{G2G3}}$ (4)

$L_{Ap_{\mathrm{eff}}}=L_{\mathrm{Ap}}\cdot\frac{E_{\mathrm{Drift}}}{E_{\mathrm{Ap}}}$ (5)

$L_{\mathrm{Drift}_{\mathrm{eff}}}=L_{\mathrm{In}j_{\mathrm{eff}}}+L_{G1G2_{\mathrm{eff}}}+L_{G2G3_{\mathrm{eff}}}+L_{\mathrm{Drift}}+L_{Ap_{\mathrm{eff}}}$ (6)

$U_{\mathrm{Drif}t_{\mathrm{eff}}}=L_{\mathrm{Drift}_{\mathrm{eff}}}\cdot E_{\mathrm{Drift}}$ (7)


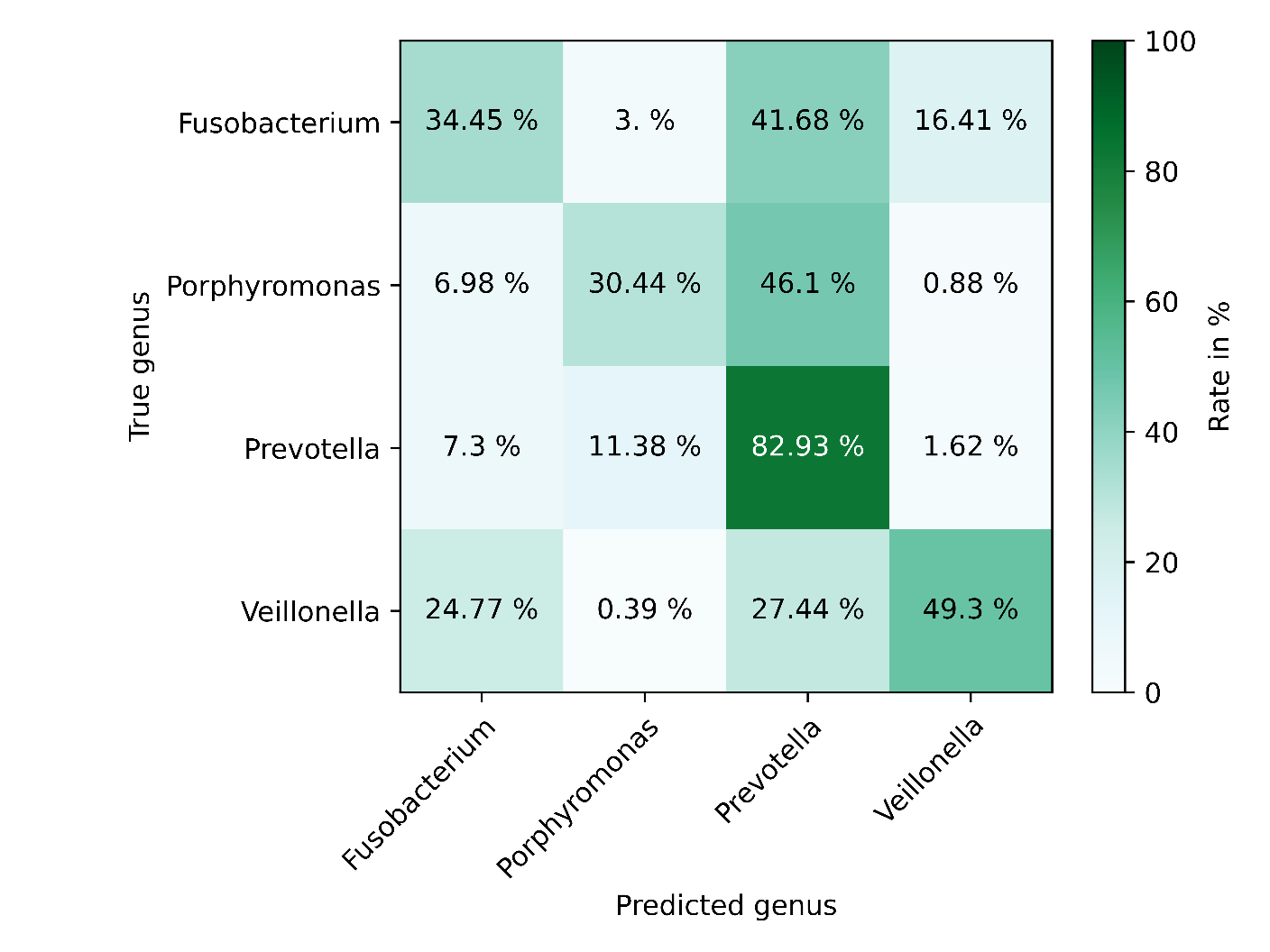


SI 1: Confusion matrix of the predicted genus by the classification model for all 95 positive peaks with SVC with a polynomial kernel of degree 2


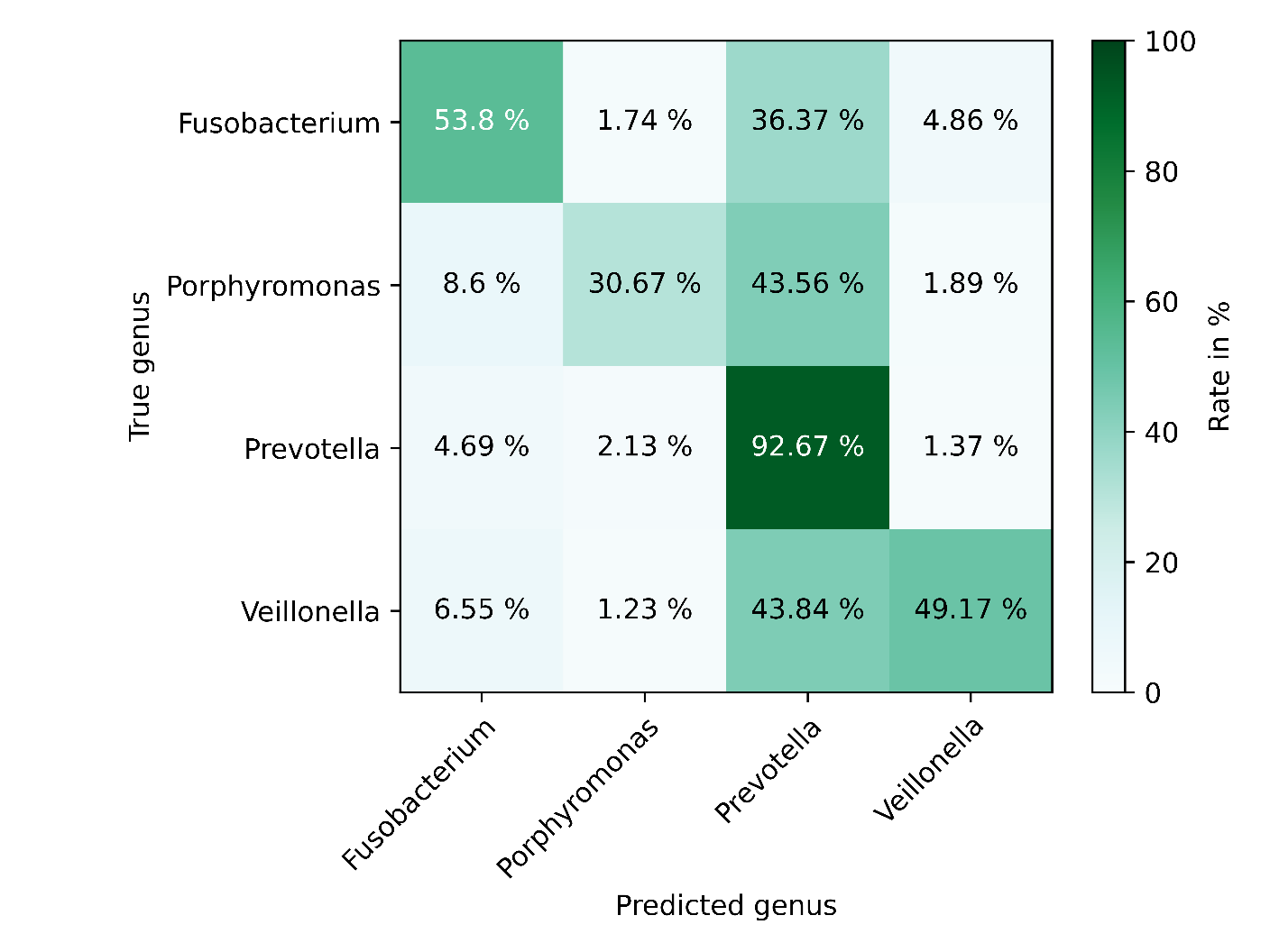


SI 2: Confusion matrix of the predicted genus by the classification model for all 95 positive peaks with SVC with a polynomial kernel of degree 3


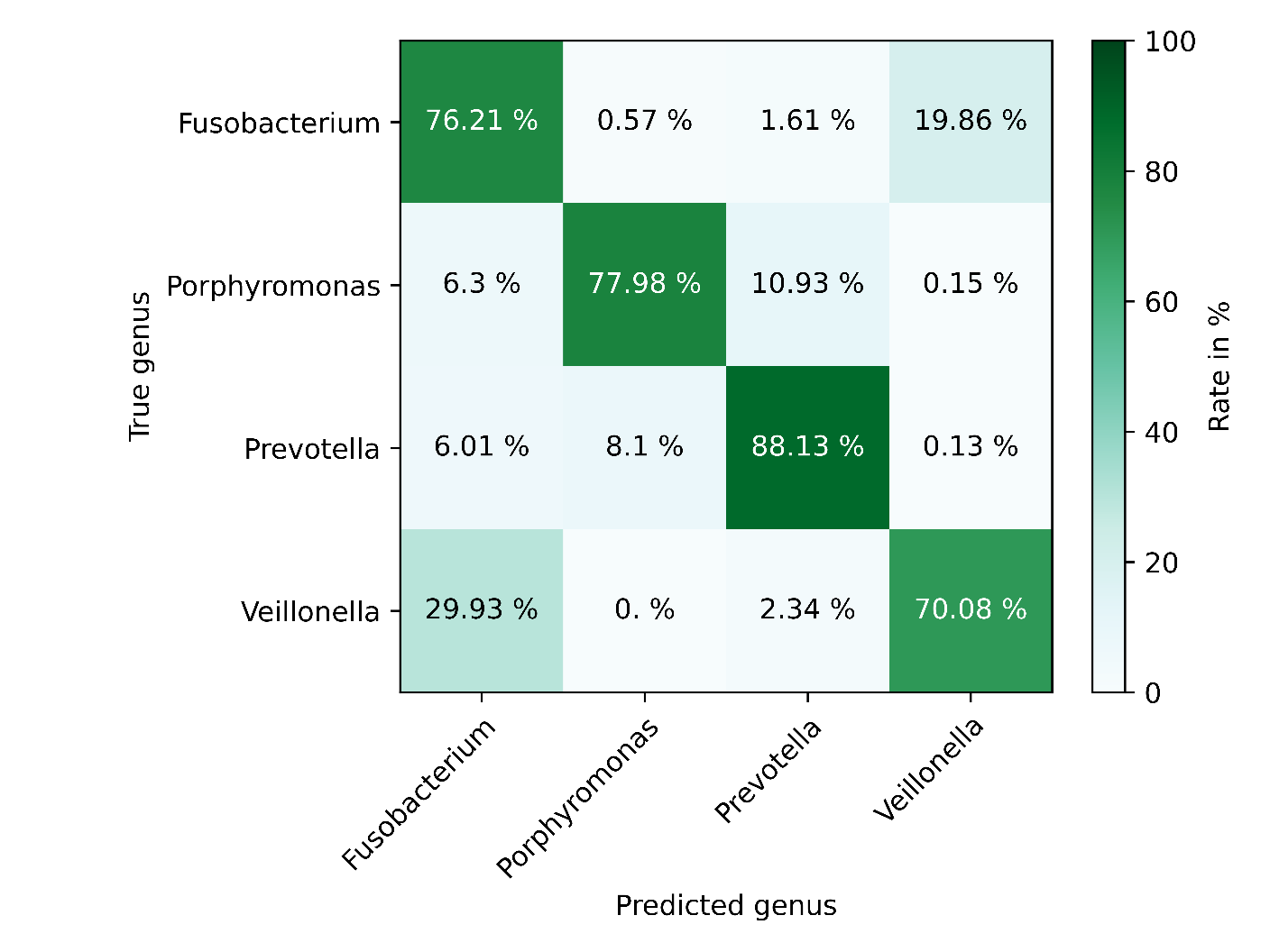


SI 3: Confusion matrix of the predicted genus by the classification model for all 95 positive peaks with SVC with a radial basis function (RBF) kernel


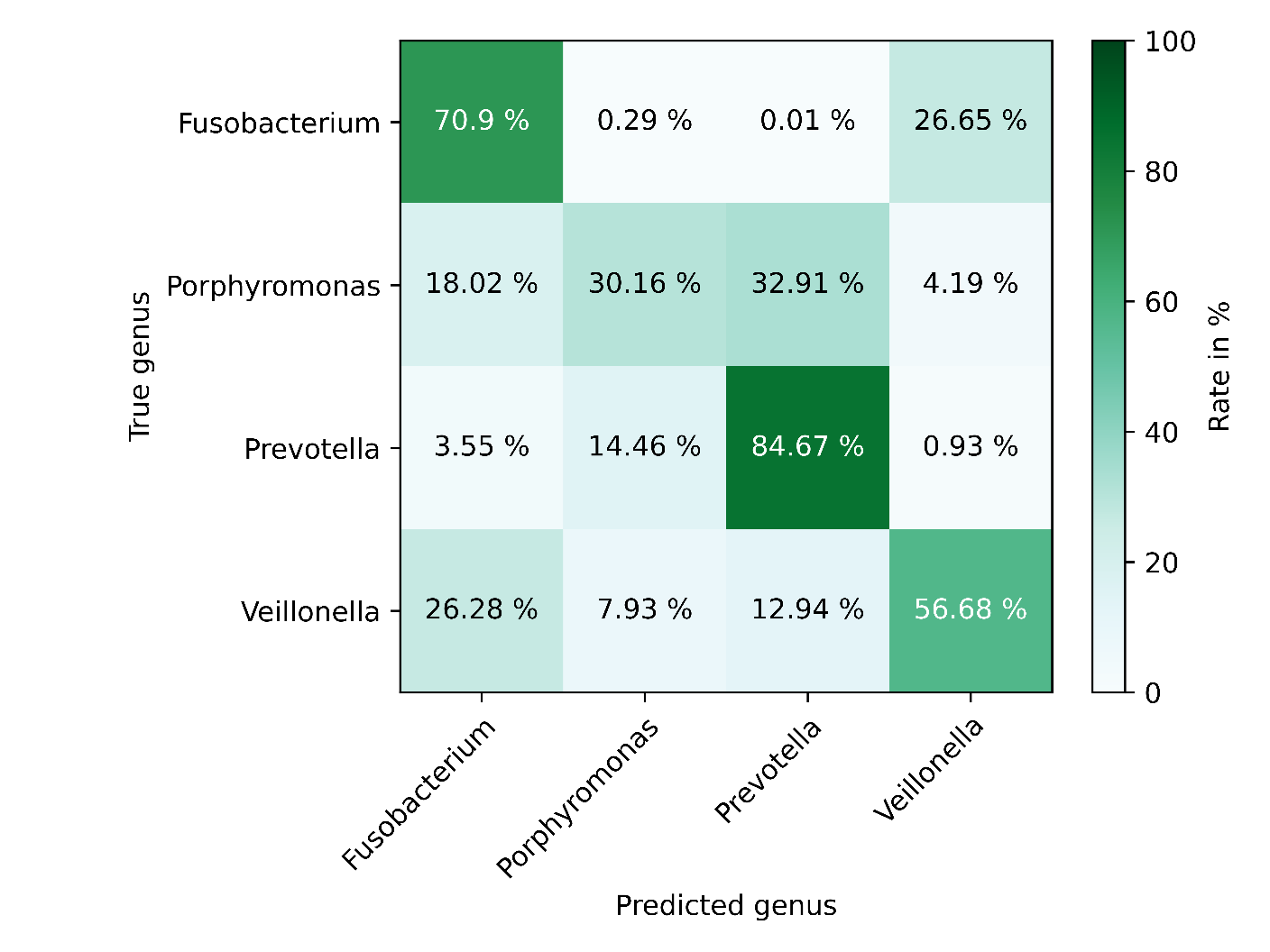


SI 4: Confusion matrix of the predicted genus by the classification model for all 95 positive peaks with SVC with a sigmoid kernel


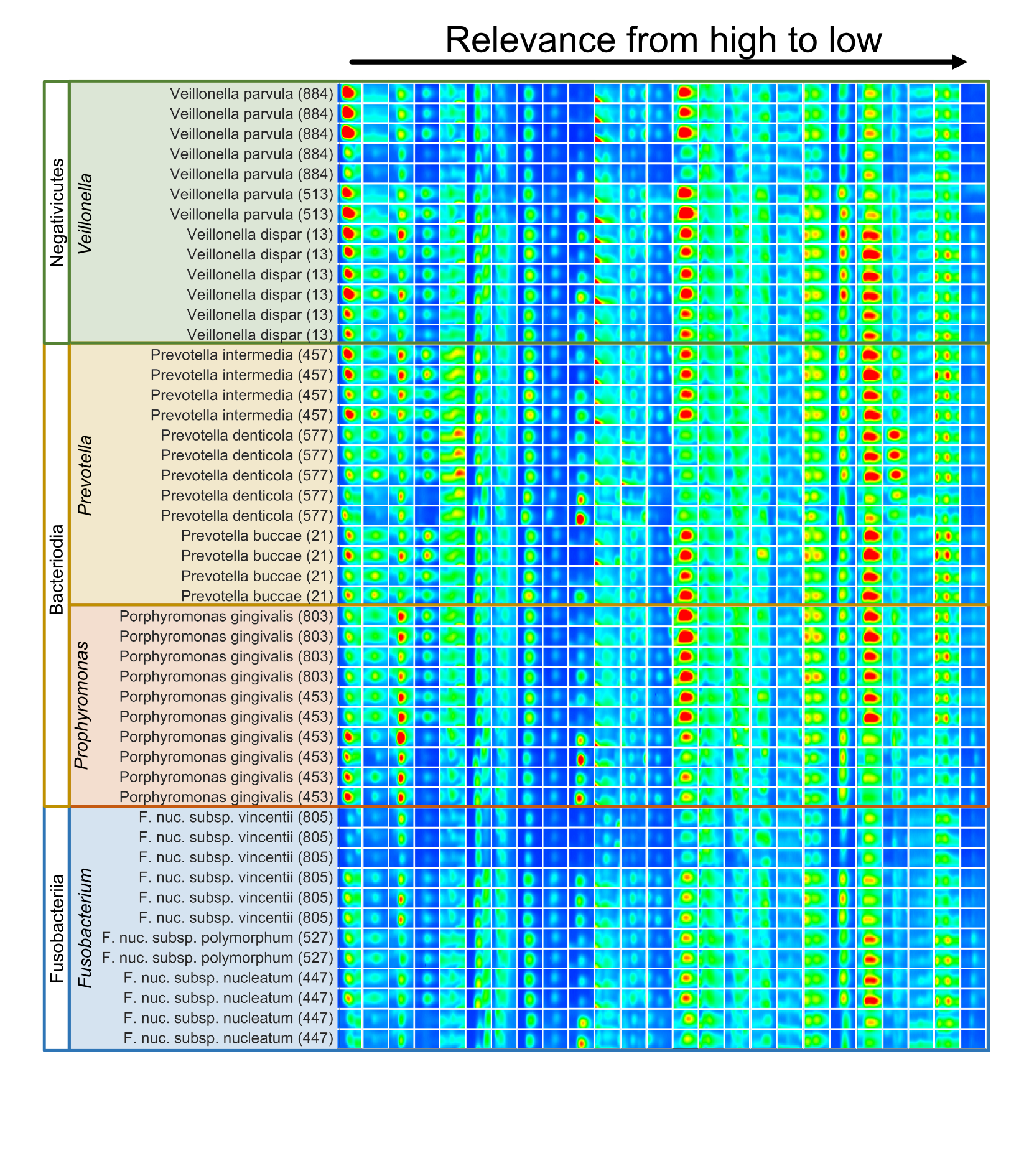


SI 5: The peaks of the model with the 25 most relevant peaks in all measurements
